# Biofilm and invertebrate consumption by western sandpipers (*Calidris mauri*) and dunlin (*Calidris alpina)* during spring migratory stopover: insights from tissue and breath CO_2_ isotopic (*δ*^13^C, *δ*^15^N) analyses

**DOI:** 10.1093/conphys/coac006

**Published:** 2022-02-18

**Authors:** Keith A Hobson, Tomohiro Kuwae, Mark C Drever, Wendy E Easton, Robert W Elner

**Affiliations:** 1 Environment and Climate Change Canada, 11 Innovation Blvd., Saskatoon, Saskatchewan, S7N 3H5, Canada; 2 Department of Biology, University of Western Ontario, 1151 Richmond St., London, Ontario, N6A 3K7, Canada; 3 Coastal and Estuarine Environment Research Group, 3-1-1, Nagase, Yokosuka 239-0826, Japan; 4 Environment and Climate Change Canada, 5421 Robertson Rd., Delta, British Columbia, V4K 3Y3, Canada

**Keywords:** staging physiology, nitrogen-15, carbon-13, Calidris mauri, Calidris alpina, breath CO2

## Abstract

Shorebirds use key migratory stopover habitats in spring and fall where body proteins are replenished and lipids stored as fuel for the remaining journey. The Fraser River estuary, British Columbia, Canada, is a critical spring stopover site for hundreds of thousands of migrating western sandpiper, *Calidris mauri*, and dunlin, *Calidris alpina*. Intertidal biofilm in spring is an important nutritional source for western sandpiper, with previous isotopic research predicting 45–59% of total diet and 50% of total energy needs. However, these studies relied on isotopic mixing models that did not consider metabolic routing of key dietary macromolecules. Complexity arises due to the mixed macromolecular composition of biofilm that is difficult to characterize isotopically. We expanded on these earlier findings by considering a protein pathway from diet to the body protein pool represented by liver tissue, using a Bayesian mixing model based on *δ*^13^C and *δ*^15^N. We used *δ*^13^C measurements of adipose tissue and breath CO_2_ to provide an estimate of the carbohydrate and protein *δ*^13^C values of microphytobenthos and used these derived values to better inform the isotopic mixing models. Our results reinforce earlier estimates of the importance of biofilm to staging shorebirds in predicting that assimilated nutrients from biofilm contribute ~35% of the protein budgets for staging western sandpipers (*n* = 13) and dunlin (*n* = 11) and at least 41% of the energy budget of western sandpiper (*n* = 69). Dunlin’s ingestion of biofilm appeared higher than anticipated given their expected reliance on invertebrate prey compared to western sandpiper, a biofilm specialist. Isotopic analyses of bulk tissues that consider metabolic routing and that make use of breath CO_2_ and adipose lipid assays can provide new insights into avian physiology. We advocate further isotopic research to better understand biofilm use by migratory shorebirds in general and as a critical requirement for more effective conservation.

## Teaser

Shorebirds feed on biofilm at their stopover site on the Fraser estuary, Canada, a phenomenon previously quantified using stable isotopes. We improve on this approach by considering metabolic routing through the novel use of breath carbon isotope analyses. Our results confirm strong protein and energy contributions of biofilm during stopover.

## Introduction

Long-distance migratory birds require stopover sites during their migrations that provide adequate nutritional benefits to maintain body condition and ultimately fuel the next leg of their journeys (Arzel *et al.*, 2006; Guglielmo, 2018; Guglielmo *et al.*, 2002; Moore & Kerlinger, 1987; Newton, 2008; Piersma, 2002). Shorebirds are among the more iconic species that accomplish this phenomenon, often aggregating in key estuaries and mudflats for days or weeks during migration (Gill *et al.*, 2009). Among these species, the western sandpiper (*Calidris mauri*) migrating along the Pacific Coast of the Americas feeds extensively on surficial biofilm in addition to invertebrate prey (Elner *et al.*, 2005; Kuwae *et al.,* 2008; Mathot *et al.*, 2010). Intertidal biofilm is an abundant and complex layer of prokaryotes, eukaryotes, meiofauna, organic detritus and sediment that forms on the surface of mudflats. The layer is suspended in a mucilaginous matrix containing carbohydrate and non-carbohydrate compounds that include proteins and lipids (Characklis, 1989; Smith & Underwood, 2000). The nutritional quality of biofilm is highly variable spatially and temporally (Schnurr *et al.,* 2020). While tracking the energetics and physiological use of biofilm is extremely challenging, this task is critical for predicting and managing the cumulative impacts of climate change, alteration of food resources, habitat destruction and disturbance at spatially limited stopover sites to prevent flyway population declines. Breakthroughs in measuring naturally occurring stable isotopes of carbon (δ^13^C) and nitrogen (δ^15^N) traced and provided the first estimates of biofilm use by western sandpipers at stopover sites on the Fraser River estuary near Vancouver, British Columbia, Canada (Beninger *et al.*, 2011; Jardine *et al.*, 2015; Kuwae *et al.,* 2008). These astonishing estimates established that biofilm provides about half of total diet and daily energy needs during spring staging *en route* to the breeding grounds.

A key advantage of measuring naturally occurring stable isotope ratios in consumer tissues is that information on assimilated, not just ingested elements, is possible. This distinction is especially important because tracing ingestion of biofilm is difficult to quantify by observation in the field (but see Elner *et al.,* 2005) or by stomach content analyses (Mathot *et al.,* 2010). Interestingly, the first application of the isotope technique to quantitatively evaluate sandpiper use of biofilm was entirely non-intrusive and relied on δ^13^C and δ^15^N measurements of the faeces of sandpipers actively foraging in the Fraser estuary as well as those of biofilm and invertebrate prey. Briefly, Kuwae *et al.* (2008) assumed the high polysaccharide content of biofilm would result in rapid metabolism of biofilm making it untraceable by conventional diet analyses, especially over the short time periods when birds were feeding at stopover sites. Further, they assumed diets, including biofilm, could be traced reasonably accurately in droppings because stomach contents of collected birds differed in biofilm- and microphytobenthos (MPB)-dominated isotopic values compared with invertebrate values in droppings, suggesting that biofilm MPB is digested to a greater extent than invertebrates. By making assumptions about the relative amount of isotopic change associated with faecal and biofilm isotope values and by employing standard isotopic mixing models, Kuwae *et al.* (2008) reconstructed sandpiper diets and concluded that their estimates of biofilm consumption were conservative and are at least 45–59% of total diet and 50% of the daily energy budget. Identical methods at more sites on the Fraser estuary and delta similarly reported that biofilm comprised between 22.8% and 53.0% of sandpiper droppings (Jardine *et al.,* 2015) and isotopic measurements of stomach contents, liver and muscle tissue of western sandpipers confirmed the relatively low sandpiper trophic levels associated with biofilm consumption and inferred a downward shift in trophic level associated with the Fraser River estuary stopover site (Beninger *et al.,* 2011).

The seminal work of Kuwae *et al.* (2008) provided foundational evidence of the nutritional significance of biofilm to migrating western sandpipers and, importantly, provided a non-intrusive means of using the stable isotope approach to evaluate dependence on this previously elusive source. Nevertheless, key assumptions (Kuwae *et al.,* 2008. Jardine *et al.,* 2015, Beninger *et al.,* 2011) require further consideration. Foremost, the interpretation of consumer diets based on Bayesian mixing models using bivariate *δ*^15^N vs. *δ*^13^C plots of consumer and dietary tissues as inputs needs consideration of metabolic routing, especially when diets can vary in macronutrient (i.e. carbohydrate, lipid, protein) composition (Podlesak *et al.*, 2005). This is particularly relevant for staging shorebirds that can convert dietary carbohydrates into stored lipids to fuel migration as well as transfer dietary lipids directly to stores. In contrast, dietary proteins are more likely to be used by shorebirds to maintain protein budgets associated with maintenance of muscle and other proteinaceous tissues (Martínez del Rio & Wolf, 2004). This is important because *δ*^15^N values in consumer and prey tissues will typically only provide information on protein source and routing, and not on lipid synthesis. Although many practitioners using stable isotope methods to reconstruct avian diets do not categorically state it, their *δ*^15^N vs. *δ*^13^C biplots typically provide a useful means of tracing protein pathways, but not lipid or carbohydrate pathways. As such, these biplots may not inherently provide an accurate picture of the biomass contributions of biofilm to the nutritional and physiological needs of staging birds. Currently, it is not clear to what extent staging shorebirds use biofilm to meet oxidative energy requirements (through lipid and carbohydrate conversions), lipid synthesis or direct storage and protein requirements. With this in mind, we performed laboratory and field studies to investigate the use of breath CO_2_*δ*^13^C measurements as well as more conventional tissue *δ*^15^N vs. *δ*^13^C and dietary assays to estimate use of biofilm by western sandpipers and dunlin (*Calidris alpina*) staging during spring in the Fraser estuary.

Although breath *δ*^13^C measurements of wild animals are uncommon (but see Podlesak *et al.,* 2005, Hobson *et al.*, 2009, Voigt, 2009, Whiteman *et al.,* 2012, Voigt *et al.*, 2013, Anparasan & Hobson, 2021), numerous laboratory studies have used this assay to monitor the uptake of carbohydrates for energy metabolism in a variety of animals (reviewed by McCue & Welch Jr., 2015). Breath CO_2_ is a byproduct of the oxidation of dietary organic compounds in animals so the *δ*^13^C measurement of breath should reflect *δ*^13^C of the substrate being metabolized. Dietary carbohydrates are typically rapidly used to produce energy to fuel flight and other exercise, so studies tracing the use of sugars in captive and wild animals often measure breath CO_2_*δ*^13^C. Fasting animals switching between metabolism of current diet vs. stored lipids typically show declines in breath CO_2_*δ*^13^C because lipids are depleted in ^13^C compared to other macromolecules (McCue & Welch Jr., 2015; Hobson and Guglielmo, unpublished data). In general, breath CO_2_ isotope measurements for small birds are expected to correspond to dietary signals based on the previous 3–6 hours (Hatch*et al.*, 2002; Podlesak *et al.,* 2005). Of course, as for all isotopic studies, the use of this approach depends on being able to accurately estimate isotopic discrimination between diets and breath and operate in a system where dietary components are well categorized.

In this study, we sought to test the results of Kuwae *et al.* (2008) and Jardine *et al.* (2015) by estimating the use of biofilm by migrating western sandpipers at the same spring stopover site by using a more conventional protein-based isotopic mixing model and exploring the use of breath CO_2_*δ*^13^C measurements. We used a small sample of collected birds to investigate the protein pathway to demonstrate a proof of principle and a larger number of individuals for non-destructive assay of *δ*^13^C in breath. We focussed primarily on western sandpipers for breath assays. Incidentally, we also captured dunlin, a larger congener, and so we report on isotopic values in dunlin, albeit with smaller (breath) sample sizes. Based on differences in functional morphology of feeding (Kuwae *et al.,* 2012), we predicted western sandpipers would show a substantial portion of their dietary macronutrients derived from direct biofilm grazing and the larger dunlin would show less direct consumption.

## Methods

### Shorebird sampling

Our study was conducted on the intertidal mudflats of Roberts Bank (49^o^05′N, 123^o^12′W) on the Fraser River estuary, British Columbia, Canada, during the northward, breeding, migration of western sandpipers (April 2016–2019). A subsample of western sandpipers (5 males, 8 females) and dunlin (7 males, 4 females) were collected (Permit SC-BC-2019-0011 from Environment and Climate Change Canada) for tissue isotope analysis according to guidelines of the Canadian Council for Animal Care, and other birds were captured using mistnets for breath analyses (69 western sandpipers, 12 dunlin) and released (see Table 2). Collected birds were kept cool in the field and transferred within 2 h to a −20°C freezer. We were interested primarily in using liver tissue as it represents a fast turnover rate tissue (Evans-Ogden *et al.*, 2004) but also opportunistically sampled pectoral muscle tissue. Sex was determined through dissection and examination of reproductive tissues. Breath sampling was conducted using a portable field device that consisted of an airtight plastic container that held the bird and could be flushed and filled with CO_2_-free air (using a hand pump to force air through a drierite and ascarite filter) before being isolated via stopcocks for 3–4 minutes allowing bird breath CO_2_ to accumulate. An air sample from the container was then subsampled through a stopcock valve via an 18-gauge needle directly into an evacuated vacutainer (Labco, Buckinghamshire, UK) and stored for later stable-carbon isotope analysis. The birds were then released.

### Food web sampling

Our mixing models relied heavily on food web components collected at our study site by Kuwae *et al.* (2008) and Jardine *et al.* (2015) and augmented by our more recent sampling to confirm isotopic consistency (Table 1). In general, biofilm was collected using a toothbrush (up to ~1 mm in depth) from various locations. MPB was extracted from sediments by modifying the method of Couch (1989); the samples were spread on a tray to ~5 mm depth, a nylon screen (65 μm mesh) was laid over the sediment and precombusted glass wool was placed over the screen. The tray was kept moist using filtered seawater and left in the dark at ~20°C overnight. The glass wool was removed and kept dry until combustion and stable isotope analysis. Small invertebrates were collected from depths of 0–2 cm in the intertidal and sorted using a 1-mm mesh sieve and large polychaetes obtained by digging (Table 1).

**Table 1:** Summary of stable isotope data available for shorebird diet samples from Roberts Bank (inter-causeway and Brunswick Point), Fraser estuary based on this study and those of Kuwae *et al.* (2008) and Jardine *et al.* (2015)

Diet	Taxa	*n*	*δ* ^15^N (‰)	*δ* ^13^C (‰)	Source
MPB		36	5.8 ± 0.7	−16.6 ± 1.9	Kuwae *et al.* (2008, 2012)
Surface sediment		9	6.8 ± 0.7	−19.5 ± 1.0	This study
		32	7.7 ± 1.7	−19.2 ± 2.6	Jardine *et al.* (2015)
		55	5.6 ± 0.7	−20.1 ± 0.9	Kuwae *et al.* (2008, 2012)
Polychaetes	Mixed	2	11.5	−11.2	This study
		2	14.0	−14.9	Jardine *et al.* (2015)
		18	12.1 ± 1.1	−15.0 ± 1.5	Kuwae *et al.* (2008, 2012)
	*Nereis*	13	11.7 ± 1.1	−15.5 ± 1.6	Kuwae *et al.* (2008, 2012)
Amphipods	*Corophium* spp.	5	8.4 ± 1.1	−11.7 ± 1.0	Kuwae *et al.* (2008, 2012)
					
Gastropods	*Batillaria attramentaria*	5	9.4 ± 1.4	−13.1 ± 2.7	This study
Bivalves	*Mya arenaria*	4	9.2 ± 1.0	−11 ± 1.2	This study
Crabs	*Hemigrapsus oregonensis*	3	8.2 ± 0.1	−14.0 ± 0.6	This study

These data, except for gastropods, bivalves and crabs, were combined to derive the best possible estimate of three trophic levels of diet available for shorebirds at this location (biofilm, small invertebrates, polychaetes) and used in the MixSiar mixing model.

### Choice of tissues

Although our focus was on using breath and liver tissues because these provided data on relatively recent diets at our stopover site, we opportunistically sampled muscle tissue. We are not sure to what degree the muscle tissue represents local feeding or feeding prior to arrival of these unmarked birds but we suspect overwhelmingly prior to arrival. Previously, *δ*^13^C and *δ*^15^N measurements of western sandpiper feathers grown at wintering sites throughout the range (Franks *et al.,* 2012) show that wintering sites are fairly constant in *δ*^13^C but do vary in *δ*^15^N. As such, we consider models based on muscle with these isotopes to be less reliable as a local tracer. We assumed that liver tissue represented dietary integrations more concordant with local feeding. Although laboratory studies to measure precisely isotopic turnover rates of various tissues in western sandpiper have not been conducted, information is available for dunlin (Evans-Ogden *et al.,* 2004; Lourenço *et al.*, 2015), our assumption that liver tissue represented local diet for sampled birds at our spring stopover location is reasonable. Evans-Ogden *et al.* (2004) estimated a half-life of 10–11 days for nitrogen and carbon turnover in whole blood of dunlin, which we assume is close to the general protein turnover rate in muscle. Lourenço *et al.* (2015) provided half-life estimates of 1.3–2.8 days for dunlin blood plasma carbon and nitrogen, respectively and 8.6–10.2 days for the cellular fraction. We assumed that liver and blood plasma have similar elemental turnover rates (Hobson & Clark, 1993). We further investigated the issue of tissue turnover rates by examining data reported for other birds. Hobson & Clark (1992) determined the isotopic half-life of 2.6 days for liver of captive (unexercised) Japanese quail (*Coturnix japonica*), and Podlesak *et al.* (2005) derived a corresponding estimate of ~1 day for Yellow-rumped warbler (*Setophaga coronate*). We know that body mass is negatively correlated (to the mass exponent of −0.25) with turnover rate (Carleton & Martinez del Rio, 2005), and migration body mass of western sandpiper is ~25 g for males and ~27.5 g for females (Franks *et al.*, 2020), whereas for dunlin, the migration body mass is ~59.7 g for males and 63.6 g for females (Warnock & Gill, 2020). Japanese quail are ~96 g (McGowan & Kirwan, 2020) and Yellow-rumped warbler are ~12.5 g (Hunt & Flaspohler, 2020). Based on these measurements, the allometric relationship reported by Carleton & Martinez del Rio (2005), and on turnover rate data from warblers and quail, we estimated isotopic half-lives for liver or blood plasma to be 1.2–1.4 days for western sandpiper and 1.5–2.3 days for dunlin. For western sandpiper, then, we suggest that a residency time on the stopover site of 2.4–2.8 days (i.e. 2 half-lives) would correspond to liver isotope values primarily representing local diet. Those values would rise to 3.0–4.6 days for dunlin. We had no way of determining when birds arrived on our site because we could not predict asymptotic isotope tissue values corresponding to residency (see Catry *et al.,* 2016; Catry *et al.*, 2017), but suggest that these data support our assumption that liver isotope data corresponded closely to local diets in western sandpiper and possibly to local diets of dunlin. Telemetry studies conducted during northward migration on the Fraser River estuary indicate stopover duration of western sandpiper and dunlin range from 1 to 4 days (Drever *et al.*, 2014, and references therein), which is consistent with this interpretation.

### Stable isotope analyses

Tissue samples from birds and invertebrates were thawed and freeze dried before being subjected to a 2:1 chloroform:methanol solvent soak and rinse to remove lipids. Samples were then ground to a powder using a mortar and pestle. Invertebrates were treated with 0.1 N HCl to remove carbonates. Lipids from subcutaneous fat samples of birds were similarly removed using the solvent treatment and isolated by evaporation in a fume hood.

For carbon and nitrogen isotope analyses, we weighed 1 mg of sample into pre-combusted tin capsules. Encapsulated samples were combusted at 1030°C in a Carlo Erba NA1500 or Eurovector 3000 elemental analyser. The resulting N_2_ and CO_2_ were separated chromatographically and introduced to an Elementar Isoprime or a Nu Instruments Horizon isotope ratio mass spectrometer (both in our laboratory). We used two reference materials to normalize the results to VPDB and AIR: BWBIII keratin (δ^13^C = −20.18, δ^15^N = +14.31‰, respectively) and PRCgel (*δ*^13^C = −13.64‰, *δ*^15^N = +5.07‰, respectively). Within-run (*n* = 5) precisions as determined from both reference and sample duplicate analyses were ± 0.1‰ for both *δ*^13^C and *δ*^15^N.

Breath samples were analysed in the Laboratory for Stable Isotope Science at Western University, London, Ontario, using a GasBench II (Thermo Fisher Scientific, Bremen, Germany) and Combi PAL autosampler (CTC Analytics AG, Swingen, Switzerland), coupled to a Thermo Finnigan Delta Plus XL isotope ratio mass spectrometer (Thermo Fisher Scientific, Bremen, Germany) operated in continuous-flow mode, with helium as the carrier gas. A calibration algorithm was based on two CO_2_ standards (−40.66‰ and −3.66‰; OzTech, Safford, USA).

### Statistical analyses

We used the R-based Bayesian mixing model MixSiar (Stock & Semmens, 2016) to estimate relative proportions of biofilm, small invertebrates and polychaetes to the diet of western sandpipers and dunlin. Dietary endpoint *δ*^13^C and *δ*^15^N data were based on our own collections and those of two previous studies conducted in our area (Jardine *et al.,* 2015; Kuwae *et al.,* 2008). The collections of Jardine *et al.* (2015) indicated the food web at our study site had not changed isotopically from the earlier study of Kuwae *et al.* (2008) and our more limited sampling was also consistent with such stability (Table 1). We restricted diet sampling and avian tissue analyses to the inter-causeway and Brunswick Point areas of Roberts Bank (see map in Jardine *et al.,* 2015). While this reduced the use of available data for the whole Fraser estuary, we noted considerable variation in isotope values based on location in the estuary (Jardine *et al.,* 2015) linked in part with the dynamics of the Fraser River plume, and so only focussed on the Roberts Bank area for all samples.

For our mixing model based on collected bird tissues, we derived the following dietary endpoints (based on the summary of data presented in Table 1): MPB (*δ*^13^C = −16.6 ± 1.9‰, *δ*^15^N = 5.8 ± 0.7‰, *n* = 36), surface sediment ‘biofilm’ (*δ*^13^C = −19.7 ± 1.7‰, *δ*^15^N = 6.4 ± 1.5, *n* = 96), small invertebrates (*Corophium*: *δ*^13^C = −11.7 ± 1.0‰, *δ*^15^N = 8.4 ± 1.1‰, *n* = 5) and polychaetes (*δ*^13^C = −15.1 ± 1.9‰, *δ*^15^N = 11.9 ± 1.2‰, *n* = 35). Kuwae (unpublished data) provided the elemental C:N ratios for these dietary endpoints for data presented in the Kuwae *et al.* (2008) study, and so we used these values as elemental concentrations in the MixSiar model (MPB, 8.1; biofilm, 9.8; small inverts, 5.1; polychaetes, 5.1). We used diet-tissue discrimination factors derived by Evans-Ogden *et al.* (2004) for captive dunlin: muscle (Δ^13^C: 1.9‰; Δ^15^N: 3.1‰) and liver (Δ^13^C: 1.1‰; Δ^15^N: 4.0‰). However, we focussed on data provided by liver samples as they were a more contemporary indicator of diet (equivalent to blood plasma) and we know that *δ*^13^C and *δ*^15^N dietary endpoints for sandpipers can vary throughout the flyway (Beninger *et al.,* 2011; Franks *et al.,* 2012).

For derivations of diet based on breath CO_2_*δ*^13^C, we considered the isotopic discrimination between dietary substrate and breath CO_2_*δ*^13^C (Δ^13^C) as 0‰ based on previous studies (reviewed by Whiteman *et al.,* 2012; McCue & Welch Jr., 2015). However, as part of a sensitivity analysis, we allowed this value to range between −1.0 and +1.0‰.

### Isotopic routing and discrimination

Using stable isotope measurements of bulk tissues to reconstruct animal diets can be complex in cases where dietary inputs can vary in macronutrient composition (e.g. Ben-David *et al.*, 2012). To date, this caution has been considered largely in terms of the various bulk dietary inputs being considered in mixing models whereby the percent available carbon and nitrogen is considered arithmetically. Such applications typically account, then, for cases where the consumer may have access to high-protein (low C:N) vs. high-carbohydrate (high C:N) foods, as in the case of bears consuming salmon versus berries (Whiteman *et al.,* 2012, see also Hobson *et al.,* 2009). To some degree, molecular routing of elements can be accounted for, and in the case of consumers primarily feeding on animal proteins, the removal of lipids from the tissues analysed results in essentially estimates of foods used in a protein pathway. For animals consuming biofilm in addition to invertebrates, as for staging shorebirds, the task of using *δ*^13^C and *δ*^15^N measurements of bulk tissues of consumer and diet becomes much more challenging. This is due to the more complex nature of biofilm that has hitherto not been considered in isotopic mixing models. The challenge primarily derives from the fact that intertidal biofilm is a community of organisms, and consists of a complex mix of proteins, lipids and carbohydrates, each potentially differing in *δ*^13^C values and associated isotopic discrimination between substrate and consumer tissue. Biofilm *δ*^15^N reflects only the protein fraction and so the bulk biofilm *δ*^15^N value is more readily quantified. However, for *δ*^13^C, we have the following mass balance equation for tissue (t) carbon *δ*^13^C derived from biofilm, *δ*^13^C_biof_(t):

*δ* ^13^C_biof_(t) = A(*δ*^13^C_carb_ + Δ^13^C_c-t_) + B(*δ*^13^C_lip_ + Δ^13^C_l-t_) + C(*δ*^13^C_prot_ + Δ^13^C_p–t_) (1)

and A + B + C = 1,

where the assimilated carbon contributions for each macromolecule are A, B and C. The isotope discrimination factors between these macronutrients and consumer tissue are depicted by the capital delta (Δ) and subscripts are carbohydrate to tissue (c-t), lipid to tissue (l-t) and protein to tissue (p-t). If the biofilm sample is pure then only these substrates need be considered. However, obtaining pure biofilm is difficult. In addition, separating the macromolecular components of biofilm and then deriving their individual *δ*^13^C values without subsequent fractionation is even more difficult and, to our knowledge, has not been attempted. Finally, deriving individual discrimination factors between each macromolecule and consumer tissue of interest has not been attempted and would require captive studies with potentially isotopically labelled or well-categorized components (e.g. Podlesak & McWilliams, 2006). Because of the difficulties described above, a goal of this study was to combine breath and tissue isotopic values to indirectly estimate the terms in equation 1 and to assess whether these estimates provided reasonable inferences.

**Figure 1:**
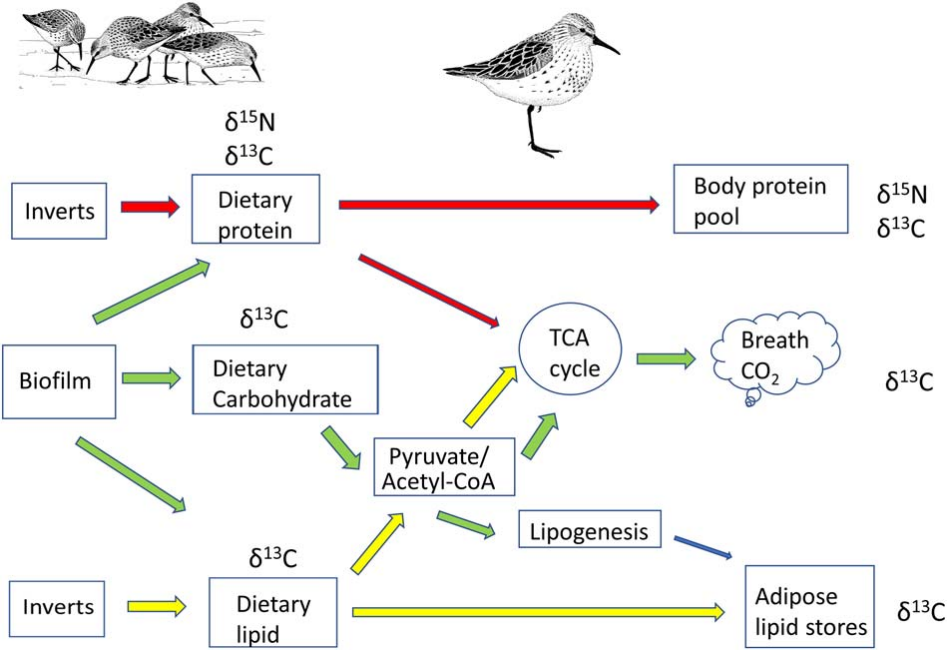
Conceptual diagram of metabolic routing of carbon and nitrogen from dietary substrates, biofilm and invertebrates to tissues of shorebirds. Biofilm contributes carbon to consumer protein, breath and lipids, whereas invertebrates contribute carbon primarily to consumer protein. Nitrogen is sourced primarily from biofilm and invertebrate protein to consumer protein. Breath CO_2_ carbon will be sourced from dietary carbohydrate during feeding and stored lipids when fasting. Figure based on Whiteman *et al.* (2012). Illustrations of western sandpiper provided by Can Stock Photo.

Consideration of macromolecular routing in the case of shorebirds feeding on biofilm and invertebrates is depicted in Fig. 1. Here, we see that the *δ*^15^N value of the body protein pool (e.g. muscle, liver, blood, feathers) can be considered a direct path from diet. However, carbon can enter three main pathways and ultimately end up in consumer protein, breath and adipose tissue. Carbon from dietary amino acids can be incorporated via the tricarboxylic acid cycle (TCA cycle) into breath CO_2_ and lipids (Newsome *et al.*, 2014), although we can consider these relatively minor in most cases.

Figure 1 illustrates the potential advantage of measuring the *δ*^13^C value of breath CO_2_ from staging shorebirds. If dietary carbohydrates are the main energy substrate for shorebirds while feeding, then the breath CO_2_*δ*^13^C values will overwhelmingly represent the *δ*^13^C value of the dietary carbohydrates, which in turn would be derived almost exclusively from biofilm. For fasting birds, we expect the metabolism of stored lipids to dominate the *δ*^13^C value of breath. While much more research is required, the consensus is that the *δ*^13^C isotopic discrimination factor between substrate and breath CO_2_ is negligible (i.e. Δ^13^C_c-t_ = Δ^13^C_l-t_ = 0; McCue & Welch Jr., 2015; Whiteman *et al.,* 2012). Based on equation 1, measuring the *δ*^13^C value of breath CO_2_ as well as the adipose tissue of migrating shorebirds can thus provide important information on the *δ*^13^C value of dietary carbohydrates and lipids.

## Results

### **Conventional dietary mixing models using δ** ^**15**^ **N and δ** ^**13**^ **C**

Tissue *δ*^15^N and *δ*^13^C values of muscle and liver of western sandpipers and dunlin showed clear marine diets for the majority of samples. However, a few individuals distinctly signalled evidence of upland terrestrial feeding both for longer-term muscle integration (western sandpipers, *δ*^13^C: −23.3‰, −22.5‰; dunlin, *δ*^13^C: −19.2‰, −20.1‰, −24.2‰) and for shorter-term liver values (western sandpipers, *δ*^13^C: −21.4‰, −23.1‰). As such, we only used those individuals without isotopic evidence of terrestrial dietary influences in our mixing models (western sandpiper: 11, dunlin 8–11; Table 2). We found no differences in muscle or liver tissue isotope values between males and females for both species (western sandpiper: liver *δ*^13^C, *F* = 0.70, *P* = 0.40; liver *δ*^15^N, *F* = 0.81, *P* = 0.38; muscle *δ*^13^C, *F* = 2.9, *P* = 0.10, muscle *δ*^15^N, *F* = 0.84, *P* = 0.38; dunlin: liver *δ*^13^C, *F* = 0.68, *P* = 0.43; liver *δ*^15^N, *F* = 1.29, *P* = 0.29; muscle *δ*^13^C, *F* = 3.1, *P* = 0.13, muscle *δ*^15^N, *F* = 0.39, *P* = 0.56) and so combined data from both sexes in tissue-specific mixing models. We present results for models based on liver and breath samples, and for completeness we provide results for muscle tissue in the Supplementary Materials (Tables S1, S2; Figs S1A and B, S2) because they represented a dietary integration period that included non-local feeding (see Discussion).

**Table 2:** Summary of shorebird tissue isotope values collected at Roberts Bank, BC (20–23 April 2016 and 30 April 2019)

Species/tissue	Date	*n*	*δ* ^15^N (‰)	*δ* ^13^C (‰)
Western sandpiper				
Muscle	20–23 April 2016	8	12.8 ± 2.7	−16.3 ± 1.1
	30 April 2019	3	11.9 ± 1.1	−15.5 ± 2.0
Liver	20–23 April 2016	8	13.7 ± 1.6	−16.0 ± 0.8
	30 April 2019	3	12.9 ± 0.5	−15.9 ± 1.1
Lipids	20–23 April 2016	8	NA	−19.7 ± 0.8
Dunlin				
Muscle	20–23 April 2016	8	12.4 ± 1.5	−15.7 ± 1.2
Liver	20–23 April 2016	11	12.9 ± 0.8	−15.6 ± 1.9
Lipids	20–23 April 2016	11	NA	−18.6 ± 1.3

**Figure 2:**
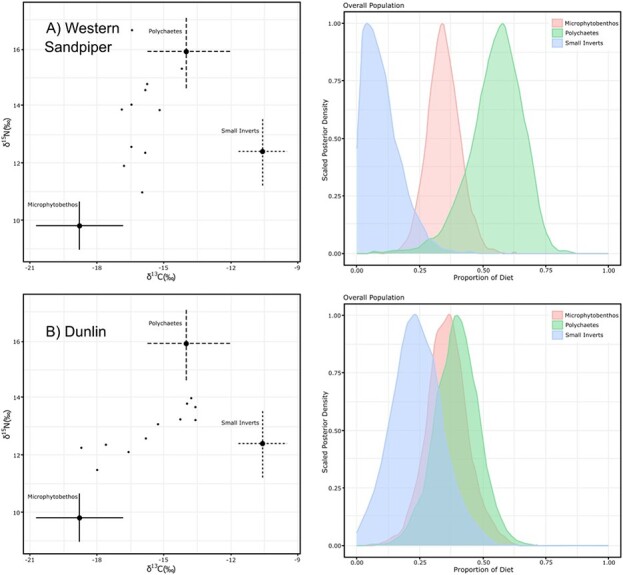
Posterior probability distributions of dietary inputs to (**A**) western sandpiper and (**B**) dunlin based on liver *δ*^13^C and *δ*^15^N measurements and using the MixSiar Bayesian mixing model. Samples were from staging shorebirds during 20–23 April 2016 and 2019 on Roberts Bank, British Columbia.

We had a choice of isotope values for representing biofilm use by shorebirds. One option was based on surface sediments carefully sampled from the top few millimetres of substrate, and the other was a purer sample of MPB derived by Kuwae *et al.* (2008). The ‘biofilm’ samples undoubtedly included inorganic sediments. As expected, the median contributions of biofilm to diets of both species were slightly higher for models based on the MPB model vs. the biofilm model. Western sandpiper showed similar median biofilm or MPB contribution to body protein (34.5–44.6%) to that of dunlin (36.5–41.6%). However, Bayesian credible intervals tended to be broad for both species (Fig. 2A and B; Supplementary Material Fig. S1A and B).

### **Breath CO**_**2**_ **model**

**Figure 3:**
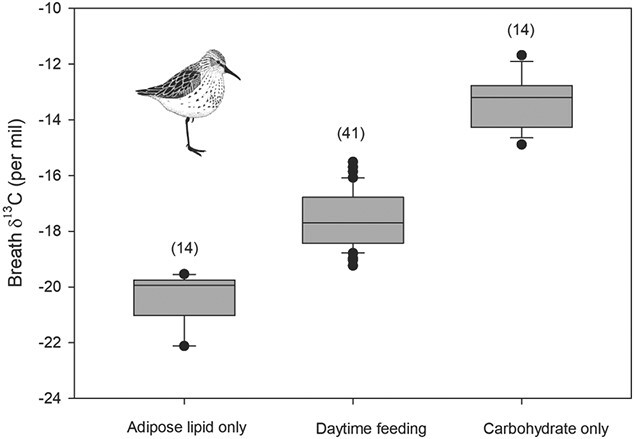
Breath CO_2_*δ*^13^C values for western sandpiper sampled during 20–23 April 2016 and 2019 on Roberts Bank, British Columbia. Birds sampled early in the morning as they returned from night roosts were assumed to be fasting and their breath representing metabolism of stored lipids. Those with the 14 most-positive (daytime) breath *δ*^13^C values were assumed to be based entirely on carbohydrate metabolism from diet. Daytime feeders were the remainder of the sample with intermediate metabolism likely representing carbohydrate and lipid metabolism

Given we captured shorebirds opportunistically, birds were sampled for breath CO_2_ at varying times of the day and tide cycles, and so birds were expected to range considerably in their extent of feeding, fasting and satiation. Our breath CO_2_*δ*^13^C results indeed showed a range of values reflecting this broad spectrum. Western sandpipers ranged from −22.1‰ to −11.7‰ (*n* = 69) and dunlin from −21.5‰ to −13.0‰ (*n* = 12). Birds with the lowest breath CO_2_ δ^13^C values were those sampled in 2019 early in the morning, captured as they returned to the mudflats from upland nocturnal roosting sites. We assumed these birds had not fed overnight on adjacent agricultural lands but could not confirm this without stomach sampling. Instead, their most negative breath CO_2_ δ^13^C values were consistent with oxidation of stored lipids. The majority of birds sampled for breath were captured throughout the day. Our sample allowed us to estimate endpoints related to empty stomachs (i.e. early morning intercepts from roost sites) and metabolizing stored adipose lipids at one extreme and assumed metabolism of biofilm carbohydrates at the other extreme. So, we arbitrarily chose the 14 most-positive (−13.3 ± 0.9‰) and 14 most-negative (−20.4 ± 0.9‰) western sandpiper samples to estimate the breath CO_2_*δ*^13^C endpoints corresponding to carbohydrate metabolism and lipid metabolism, respectively (Fig. 3). Although this choice was arbitrary, the relatively narrow SD for each of these groups suggests the individuals within each were indeed using similar mixtures of oxidative substrates. Then, we calculated the relative amount of biofilm used as metabolic fuel using a one-isotope (*δ*^13^C) and two-endpoint (pure carbohydrate vs. pure lipid metabolic substrate) linear model on the remaining intermediate samples. For western sandpiper the mean contribution of biofilm carbohydrate to energy metabolism was 40.7 ± 14.4% (*n* = 41). Our sample of dunlin was too small (*n* = 12) to derive a reliable estimate here but was highly variable (i.e. 68.5 ± 43.9%; *n* = 12).

### Insights into biofilm isotopic composition

Our derivation of the *δ*^13^C value of adipose tissue of western sandpiper and dunlin together with *δ*^13^C values of MPB provides us with a means of estimating the *δ*^13^C values of key macromolecular components. The *δ*^13^C value of breath CO_2_ for presumed fasting western sandpipers (−20.4 ± 0.9‰) corresponds to the same predicted adipose tissue value (i.e. with no isotopic discrimination), and this value is in excellent agreement with our measured adipose lipid samples (western sandpiper: −19.7 ± 0.8‰; dunlin:−18.6 ± 1.3‰). The breath sample *δ*^13^C value corresponding to presumed full carbohydrate metabolism (−13.3 ± 0.9‰) provides a first estimate for biofilm carbohydrate. If we assume a biofilm lipid *δ*^13^C value of −20‰ based on our fasting breath *δ*^13^C value and that there is little isotopic change in lipids as they are transferred up the food web due to little remodelling isotopically in an otherwise carnivorous consumer (Polischuk *et al.*, 2001, but see Budge *et al.*, 2011), then we can back calculate the protein δ^13^C value of MPB by mass balance from the relationship:

*δ* ^13^C_MPB_ = A(*δ*^13^C_carb_) + B(*δ*^13^C_lip_) + C(*δ*^13^C_prot_), (2)

where A, B and C are the proportion of carbon from carbohydrate, lipid and protein available in MPB, respectively. Assuming A = 0.5, B = 0.1 and C = 0.4, from samples collected at study site and analysed (following Mogle, 2021; Drever MC, unpublished data; see also Pusceddu*et al.*, 1999), then

*δ* ^13^C_prot_ = [*δ*^13^C_MPB_—A(*δ*^13^C_carb_)—B(*δ*^13^C_lip_)]/C (3)

or

*δ* ^13^C_prot_ = [−16.6_−_0.5(−13.3)—0.1(−20)]/0.4 = −19.9‰.

This *δ*^13^C estimate for the protein fraction of MPB is considerably lower than the measured value for bulk material (−16.6 ± 1.9‰) due to the presence of the enriched carbohydrate fraction (i.e. −13.3‰) and provides a better estimate of the protein dietary component used by shorebirds to form body proteins. Therefore, we considered a plausible protein dietary endpoint for MPB to be −19.9 ± 1.9‰ for *δ*^13^C and 5.8 ± 0.7‰ for *δ*^15^N. Using these values resulted in measured liver tissue values falling well within the mixing polygon (Fig. 2). We present the liver-based mixing model results using this isotopic endpoint as MPB-protein in Table 3. We then considered the effect of varying the diet-breath isotopic discrimination factor Δ^13^C from an assumed value of 0‰ to −1.0 and +1.0‰ (for Δ^13^C = −1.0‰: *δ*^13^C_carb_ = −12.3‰, *δ*^13^C_lip_ = −19.4‰ and *δ*^13^C_prot_ = −21.4‰; for Δ^13^C = +1.0‰: *δ*^13^C_carb_ = −14.3‰, *δ*^13^C_lip_ = −21.4‰ and *δ*^13^C_prot_ = −18.4‰). Under these scenarios, estimates of the MPB contribution varied for western sandpipers (29–41%) and dunlin (31–42%). As expected, for both species, the lowest estimate of MPB *δ*^13^C_prot_ based on the results using Δ^13^C = −1‰ resulted in a lower estimate of biofilm (MPB) protein contribution to diet and the higher MPB *δ*^13^C_prot_ estimate using Δ^13^C = +1‰ resulted in a higher biofilm contribution compared to using Δ^13^C = 0‰ (Supplementary Material Table S2).

**Table 3:** Estimated contributions of surface sediments (BIOF) or MPB, small invertebrates and large polychaetes in western sandpiper and dunlin liver tissue for birds collected at Roberts Bank, British Columbia (20–23 April 2016 and 30 April 2019)

Species (model)	Biofilm/MPB		Small inverts		Polychaetes	
	Median	95% Cr. I.	Median	95% Cr. I.	Median	95% Cr. I.
Western sandpiper (BIOF)	34.5%	6.6–48.1%	13.6%	8.5–33.0%	51.9%	11.7–71.8%
Western sandpiper (MPB)	44.6%	13.1–70.3%	4.3%	4.3–16.2%	51.0%	13.8–77.9%
Western sandpiper (MPB-protein)	34.5%	6.4–47.5%	10.1%	7.5–27.7%	55.4%	10.5–72.6%
						
Dunlin (BIOF)	36.5%	8.7–54.5%	30.4%	9.9–50.1%	33.1%	9.0–51.0%
Dunlin (MPB)	41.6%	8.6–56.6%	8.6%	7.8–29.4%	49.8%	7.2–63.5%
Dunlin (MPB-protein)	36.1%	8.2–53.2%	24.2%	10–45.3%	39.6%	8.6–56.2%

Data presented are median inputs based on 3-source MixSiar Bayesian mixing models and 95% credibility intervals.

## Discussion

Our study used *δ*^13^C and *δ*^15^N measurements of shorebird tissues to evaluate the diets of spring staging western sandpipers and dunlin at Roberts Bank on the Fraser River estuary. In contrast to previous studies, we considered as much as possible metabolic routing of bulk macromolecules in our isotopic mixing models and, for the first time, report on the use of breath CO_2_*δ*^13^C measurements to estimate sources of energy metabolism in shorebirds during a migratory stopover. Our results not only confirm the importance of biofilm for energy metabolism as well as a source of protein, manufactured and assimilated lipids to western sandpipers, but also reveal the importance of biofilm to dunlin, a species previously assumed to be more dependent on invertebrates (Kuwae *et al.,* 2012). Our study paves the way for future more refined approaches using bulk and compound-specific isotope techniques. Below, we consider our results for both species based on liver, adipose tissue and breath CO_2_ but refer the reader to Supplementary Material for results associated with muscle tissue that inherently corresponded to diets prior to arrival on Roberts Bank (Beninger *et al.,* 2011) and which may involve different but generally unknown isotopic baselines (Franks *et al.,* 2012).

Kuwae *et al.,* 2008 pioneered isotopic studies of staging western sandpiper at our study site using a non-invasive approach of measuring the isotopic composition of droppings to represent sandpiper diets, a study followed by Kuwae *et al.* (2012). That work, together with subsequent isotopic studies (Jardine *et al.,* 2015) assumed the undigested organic matter in the droppings (following treatment for metabolites and carbonates) resembled isotopically that of the diet. The digestion of MPB in the process provided a conservative estimate of biofilm use. The isotope mixing model used by Kuwae *et al.* (2008) and Jardine *et al.* (2015) assumed that the *δ*^13^C value of the dietary components represented either primarily a protein pathway (based on the use of *δ*^15^N) or that the *δ*^13^C value of whole droppings, as well as the *δ*^13^C value of all dietary endpoints reflected the use of all dietary macromolecules contributing to consumer protein. That assumption was also made by Beninger *et al.* (2011) using more direct tissue (liver, muscle) isotope assays. We assessed this major assumption by better consideration of macromolecular routing. We first ran mixing models based on shorebird proteinaceous tissues using both surface sediment ‘biofilm’ and MPB as basal dietary inputs and considered short-term integration of diet provided by liver isotopic assays. Those concentration-dependent Bayesian models estimated that biofilm contributed 34.5–44.6% of the diet *leading to body proteins* of western sandpiper and 36.5–41.6% of the diet of dunlin. Those estimates confirm the findings of the previously mentioned studies based on droppings that reported median values of 36.4–37.7% (Jardine *et al.,* 2015) and 45–59% (Kuwae *et al.* (2008) biofilm to the ‘whole’ diet.

Our derivation of the contribution of biofilm to the body protein pool of dunlin is new. We had assumed that this dietary endpoint would contribute much less protein to individual dunlin diets because this larger species has less developed tongue spines associated with biofilm feeding (Elner *et al.,* 2005; Kuwae *et al.,* 2012) and appears overall more adapted to probe for infaunal invertebrate prey (Elner *et al.,* 2005). Nonetheless, dunlin likely consumed substantial biofilm at our study site. While dunlin may be able to satisfy their stopover protein demands through feeding on invertebrates, our estimate that biofilm contributed at least a third of their protein body pool is important and indicates a preference for biofilm by staging shorebirds in general. We encourage further studies to evaluate diets isotopically of more shorebird species staging during migration.

For both western sandpiper and dunlin, small invertebrates contributed little to protein budgets during spring stopover. Instead, polychaetes and presumably similar trophic-level larger invertebrate prey contributed approximately half of dietary protein. Staging shorebirds require protein to rebuild muscle mass for the next long-distance flight (Guglielmo *et al.*, 2001; Piersma, 2002), and these needs at Roberts Bank are provided by biofilm protein as well as by invertebrates. The other major component of biofilm is carbohydrate bound in the extracellular polymeric substances, and this dietary component can be used to synthesize lipids for storage as adipose tissue to fuel subsequent migration and be readily available for oxidative energy production. We derived an estimate of the *δ*^13^C value of the metabolic substrate being used directly for energy using breath CO_2_*δ*^13^C measurements. We reasoned that birds sampled following an overnight fast as they arrived on the mudflats would be metabolizing stored lipids and so provide an estimate of adipose *δ*^13^C. Similarly, we assumed that the most enriched breath CO_2_*δ*^13^C measurements of foraging birds later in the day would provide an estimate of the dietary (biofilm) carbohydrate *δ*^13^C value. These assumptions were supported by our simple linear model and confirmed isotopically use of adipose tissue and biofilm carbohydrates representing fasting and feeding, respectively. Moreover, the model predicted a convincing estimate of biofilm protein *δ*^13^C as −19.9‰. We then used this model to predict the relative use of carbohydrate vs. adipose tissue or dietary lipids for energy production and estimated foraging western sandpipers derived approximately 40.7% of energy from biofilm carbohydrate. Our estimate for dunlin was actually higher, but based on relatively fewer individuals. A previous non-isotopic energetics model (Kuwae *et al.,* 2008), estimated western sandpipers derived approximately 50% of their daily energy budget from biofilm. Our data based on a breath isotope model supports this estimate, especially considering that our individual data were derived from a single sampling point during the day.

Finally, by using estimates of biofilm isotopic endpoints corresponding to biofilm carbohydrates and lipids (based on our breath model), we obtained an estimate of the biofilm protein *δ*^13^C value (i.e. −19.9‰) that, combined with the MPB *δ*^15^N value (5.8 ± 0.7‰), gave us our best estimate of the protein fraction of biofilm used by shorebirds. This model (MPB-protein) predicted ~34.5% and 36.1% median protein contributions to western sandpipers and dunlin, respectively.

We identified a few collected western sandpiper individuals with clear terrestrial protein diets (based on liver and muscle tissues), obtained either in the vicinity or *en route*. As our breath sample model was designed to approximate oxidation of stored lipids vs. carbohydrates and assumed that early morning returning birds were metabolizing only stored lipids during an overnight fast, it is possible that some individuals were also metabolizing terrestrial diets. We assume that this was a relatively small source of error for our lipid isotopic endpoint in the breath mixing model that was instead generally consistent with direct oxidation of adipose tissue. Nonetheless, previous isotopic investigations have used stable isotopes to document terrestrial foraging by wintering dunlin either nocturnally or during high-tide cycles (Evans-Ogden *et al.*, 2005; Hobson *et al.*, 2013) and these records can now be expanded to include western sandpiper that may forage terrestrially especially at times when high tides force them ashore.

Beninger *et al.* (2011) used *δ*^13^C and *δ*^15^N measurements of stomach contents, liver and muscle tissue of spring staging western sandpipers at our study site and, by contrasting isotopic mixing model predictions based on these assumed differing periods of dietary integration, predicted a downward shift in trophic position during migration (see also Hall *et al.*, 2021). Although we generally agree that these different tissues and components clearly represent different periods of integration with muscle almost certainly reflecting diet prior to arrival at our site, the absence of ground-truthed isotopic baseline data along the flyway and the lack of knowledge of individual movement history remain problematic. Indeed, our own comparison of isotopic model results using liver and muscle tissue for both western sandpiper and dunlin provides no such evidence based simply on the locally well-categorized isotopic food web at our single site. To move forward, studies at key stopover and wintering sites along the flyway (e.g. studies quoted here for the Fraser estuary; Hall *et al.,* 2021, for San Francisco Bay) that establish tissue isotope values of birds in isotopic equilibrium with local food webs are needed.

###  

#### Future work

Our preliminary study clearly demonstrates the benefits of using shorebird and food web stable isotope measurements in a concentration-dependent Bayesian mixing model framework to estimate shorebird use of biofilm and invertebrate prey during migratory stopover. This approach clearly allows information on individual macronutrients vs. bulk diet. The inclusion of breath sampling to allow estimates of which substrate is being used for oxidative energy production adds both a valuable component on its own but also can inform the isotopic composition of biofilm macromolecules, as we have demonstrated. Nonetheless, there are several research areas that can still be improved to better inform stable isotope modelling. For example, although we chose to use tissues of collected birds to allow sampling of the body protein pool and adipose lipids, future studies can use plasma and cellular blood fractions to represent rapid and slower tissue dietary protein assimilation (Hobson & Clark, 1993). Potentially, adipose tissue could also be sampled non-destructively via biopsy (Rocha *et al.*, 2016). Real-time breath δ^13^C analysis can now be achieved using laser-based instruments in the field that also avoid issues of rebreathing (Voigt, 2009; see also Mitchell *et al.*, 2015). Such improvements will result in the non-destructive sampling of large numbers of individuals and so open up numerous research directions.

Biofilm is chemically complex (Ollinik *et al.,* 2021) and difficult to categorize isotopically. To our knowledge, biofilm has never been separated into protein, lipid and carbohydrate fractions for isotopic analysis. Indeed, our approach of using breath analyses to estimate the carbohydrate δ^13^C value, based on the assumption that a high carbohydrate diet will overwhelmingly be used for energy metabolism vs. proteins or lipids, might be the only practical way of evaluating the importance of carbohydrates to sandpipers in natural settings. To evaluate the isotopic discrimination values between dietary macromolecules and shorebird tissues, including breath, will require captive rearing of shorebirds on artificial diets with known (and possibly labelled) macromolecules (see Hobson & Clark, 1993; Podlesak & McWilliams, 2006). The additional use of the respiratory quotient (RQ) as a means of confirming which macronutrients are being metabolized to CO_2_ will fundamentally advance these captive studies. If we consider only carbohydrates and fats, an RQ of 1 indicates carbohydrate metabolism whereas an RQ of 0.7 indicates pure lipid metabolism (Brody, 1999). That assumption is likely valid for sandpipers using biofilm, however, we recognize that birds oxidizing proteins for energy present a challenge because pure protein catabolism also results in an RQ of 0.7 and some amino acids are immediately oxidized without becoming available for metabolism (Wu, 1998). Knowing the δ^13^C value of the carbohydrate fraction of the diet and confirmation of carbohydrate oxidation via RQ ratio would allow more precise derivation of isotopic discrimination factors between diet and breath to be applied for sandpipers. As such, we acknowledge that our estimates of metabolic routing of biofilm macronutrients to shorebirds at our study site can be modified once such careful captive studies are conducted.

More recent advances in using compound-specific isotope approaches to tracing diet may well prove important to understanding the diets of shorebirds in general and their use of biofilm in particular (Whiteman *et al.*, 2019). That approach provides isotopic measurements of individual amino acids and fatty acids. Should biofilm have essential amino acids and fatty acids that differ isotopically from other sources of primary production, then the compound-specific isotope approach holds considerable promise as a means of tracing biofilm use. Especially intriguing is the possibility of tracing the use of essential fatty acids transported directly from biofilm (Schnurr *et al.*, 2019) vs. those that can be converted from precursor substrates (Twining *et al.,* 2016; Twining *et al.*, 2016; but see Dick & Guglielmo, 2019). Experimentally deriving conversion efficiencies from ALA to EPA by shorebirds for example would improve understanding of the overall nutritional benefits of biofilm to staging shorebirds in general and use of long-chain polyunsaturated fatty acids such as the Omega-3 components, in particular (Schnurr *et al.,* 2020). Recent research also indicates that individual fatty acids differ in their rate of oxidation relative to exercise (Carter *et al.*, 2019) and this has potential applications for the use of compound-specific approaches to investigate bird energetics during stopover. Finally, using compound-specific isotope measurements of amino acids in various shorebird tissues can potentially allow an estimate of trophic level that is independent of food web baseline. This is because within an animal there are ‘source’ amino acids that change little with trophic level (e.g. phenylalanine) and ‘trophic’ amino acids that show stepwise enrichment with trophic level. Using isotopic differences between source and trophic amino acids thus provides an estimate of trophic level without the need to sample associated food webs (reviewed by Whiteman *et al.*, 2019). That approach was first applied by Gomez *et al.* (2018) to a migratory bird and we recommend the downward trophic shift hypothesis of Beninger *et al.* (2011) to be tested this way.

In conclusion, although effective conservation of long-distance migratory birds requires the protection of key stopover as well as breeding and wintering sites (Moore & Kerlinger, 1987), few studies have linked the use of these sites to their potential for providing crucial nutritional resources (Guglielmo, 2018). Accordingly, the dietary quality of sites should be considered in addition to dietary quantity (Twining et al., 2016a, b) in order to be viewed spatially and temporally as ‘nutritional landscapes’ (Bumelis *et al.*, 2021). Complementary understanding of avian physiological needs during stopover and refueling is also lacking, particularly the roles of dietary macromolecules in meeting current and future energetic and protein requirements. The realization of intertidal biofilm being not only a source of energy (quantity) but also long-chain highly unsaturated fatty acids, such as Omega-3 (quality), essential to shorebirds on long-distance migration (Guglielmo, 2018; Young *et al.*, 2021) has opened new insight on the ecological mechanisms underpinning stopover site functioning and, when they fail, a plausible hypothesis to explain population declines in migratory shorebird species worldwide (Mathot *et al.*, 2018; Studds *et al.,* 2017; Wilson *et al.*, 2011). In doing so, recent studies into western sandpiper use of intertidal biofilm in the Fraser River estuary offer hope on how deeper appreciation of physiological requirements can boost understanding of shorebird migration generally and better inform conservation (Schnurr et al., 2018, Schnurr *et al.,* 2020; Canham*et al.*, 2021). In summary, given rapidly increasing anthropogenic threats, isotopic tissue and breath methodology offers a critical new tool to understand how spatio-temporal patterns in the biofilm ‘landscape’ align with the physiological demands of migrating shorebirds and a means to more effective conservation and restoration for stopover sites globally (Kuwae *et al.*, 2021).

## Funding

This work was funded by an operating grant to K.A.H. from ECCC and a Discovery grant to K.A.H. from the Natural Sciences and Engineering Research Council. T.K. was supported by Grants-in-Aid for Scientific Research grant numbers 18H04156 and19K20500 from the Japan Society for the Promotion of Science.

## Supplementary Material

SUPPLEMENTARY_MATERIAL_coac006
