# Supplementary material for: Biofilm and invertebrate consumption by western sandpipers (Calidris mauri) and dunlin (Calidris alpina) during spring migratory stopover: insights from tissue and breath CO2 isotopic (δ13C, δ15N) analyses
Source: Conserv Physiol. 2022 Feb 18;10(1):coac006. doi: 10.1093/conphys/coac006 (PMC8857455; doi:10.1093/conphys/coac006)
Supplement: SUPPLEMENTARY_MATERIAL_coac006 [file supplementary_material_coac006.docx]

**SUPPLEMENTARY MATERIAL**

**Biofilm and invertebrate consumption by Western Sandpipers (*Calidris mauri*) and Dunlin (*Calidris alpina)*** **during spring migratory stopover: Insights from tissue and breath CO_2_ isotopic (*δ*^13^C, *δ*^15^N) analyses**

Keith A. Hobson^1,2*^, Tomohiro Kuwae^3^, Mark C. Drever^4^, Wendy E. Easton^3^, and Robert W. Elner^4^

^1^Environment and Climate Change Canada, Saskatoon, Saskatchewan, Canada.

^2^Department of Biology, University of Western Ontario, London, Ontario, Canada.

^3^Coastal and Estuarine Environment Research Group, Nagase, Yokosuka, Japan.

^4^Environment and Climate Change Canada, Delta, British Columbia, Canada.

*Direct all correspondence to Dr. Keith Hobson, Department of Biology, University of Western Ontario, London, Ontario, Canada, N6A 5B7

Email: [khobson6@uwo.ca](mailto:khobson6@uwo.ca)

Table S1. Estimated contributions of surface sediments (BIOF)) or microphytobenthos (MFB), small invertebrates and large polychaetes in Western Sandpiper and Dunlin muscle tissue for birds collected at Roberts Bank, BC 20-23 April 2017 and 30 April 2019. Data presented are median inputs based on 3-source MixSiar Bayesian mixing models and 95% credibility intervals.

| Species (model) | Biofilm/MFB | | Small inverts | | Polychaetes | |
| --- | --- | --- | --- | --- | --- | --- |
|  | Median | 95% Cr. I. | Median | 95% Cr. I. | Median | 95% Cr. I. |
| Western (BIOF) | 50.7% | 8.5-68.9% | 10.0% | 8.4-31.5% | 39.3% | 12.8-60.5% |
| Western (MFB) | 65.6% | 16.3-92.5% | 6.6% | 6.6-24.1% | 27.9% | 16.5-65.7% |
|  |  |  |  |  |  |  |
| Dunlin (BIOF) | 41.4% | 7.5-56.4% | 12.0% | 8.0-30.6% | 46.6% | 9.1-63.0% |
| Dunlin (MFB) | 38.0% | 11.8-61.2% | 6.8% | 7.0-26.1% | 55.2% | 12.2-78.1% |
|  |  |  |  |  |  |  |

Table S2. Estimates of the contribution of microphytobenthos (MFB), small invertebrates and large polychaetes to Western Sandpiper liver tissue for birds collected at Roberts Bank, BC, 20-23 April 2017 and 30 April 2019 considering breath CO2 Δ^13^C values ranging from -1 to +1 ‰. Data presented are median inputs based on 3-source MixSiar Bayesian mixing models and 95% credibility intervals.

| Species (model) | Δ^13^C (‰) | Biofilm/MFB | | Small inverts | | Polychaetes | |
| --- | --- | --- | --- | --- | --- | --- | --- |
|  |  | Median | 95% Cr. I. | Median | 95% Cr. I. | Median | 95% Cr. I. |
| Western (MFB) | -1 | 28.6% | 5.7-40.4% | 16.2% | 9.7-38.0% | 55.2% | 12.4-76.2% |
|  | 0 | 34.5% | 6.4-47.5% | 10.1% | 7.5-27.7% | 55.4% | 10.5-72.6% |
|  | +1 | 41.4% | 8.4-58.3% | 5.8% | 5.3-19.6% | 52.8% | 10.2-71.2% |
|  |  |  |  |  |  |  |  |
| Dunlin (MFB) | -1 | 30.6% | 7.2-45.7% | 34.3% | 9.854.7% | 35.1% | 9.7-53.9% |
|  | 0 | 36.1% | 8.2-53.2% | 24.2% | 10-45.3% | 39.6% | 8.6-56.2% |
|  | +1 | 42.0% | 8.4-57.7% | 13.4% | 8.9-33.5% | 44.7% | 7.7-59.8% |

Figure S2 A. Depiction of the distribution of Western Sandpiper tissue stable isotope values relative to dietary endpoints. Data are output from the MixSiar Bayesian models used to predict posterior probability distributions.


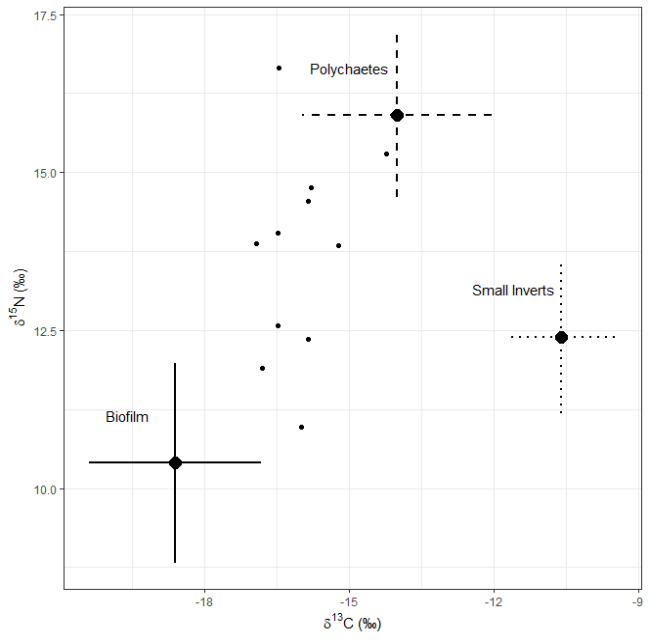

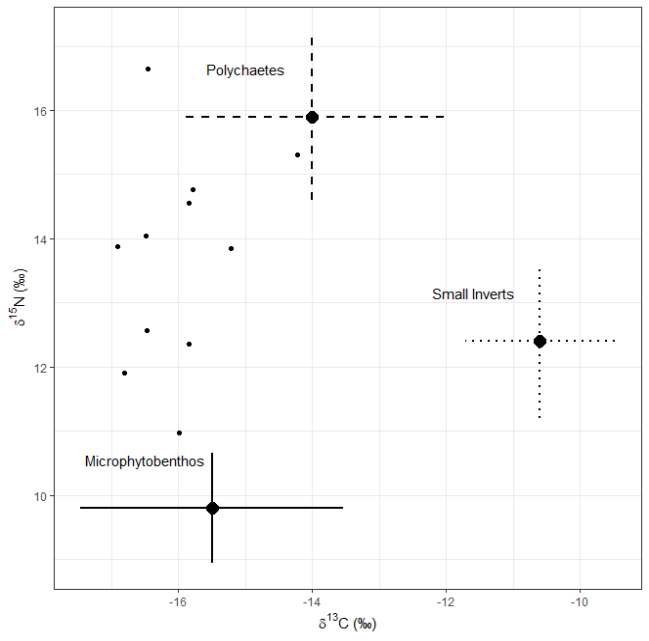

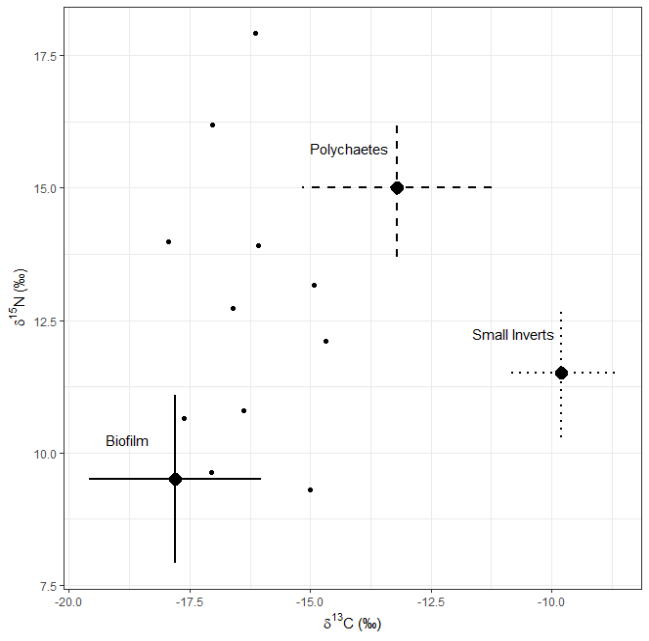

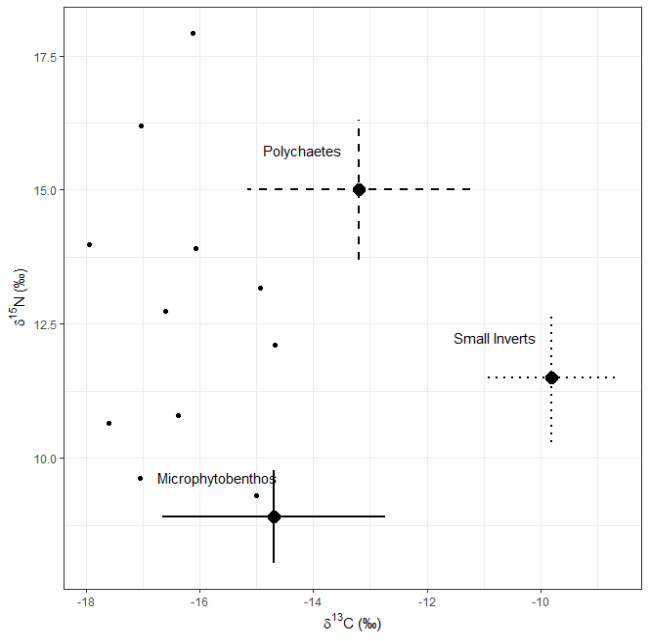


WESA – Liver- Biof

WESA-Liver-MPB

WESA-Muscle-Biof

WESA-Muscle-MPB

Figure S1 B. Depiction of the distribution of Dunlin tissue stable isotope values relative to dietary endpoints. Data are output from the MixSiar Bayesian models used to predict posterior probability distributions.


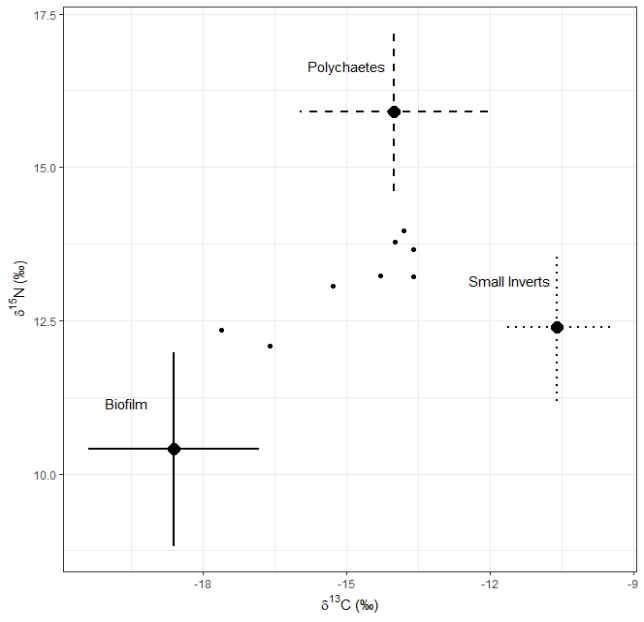


DUNL-liver-Biof

DUNL liver-MPB

DUNL-muscle-Biof

DUNL-muscle-MPB


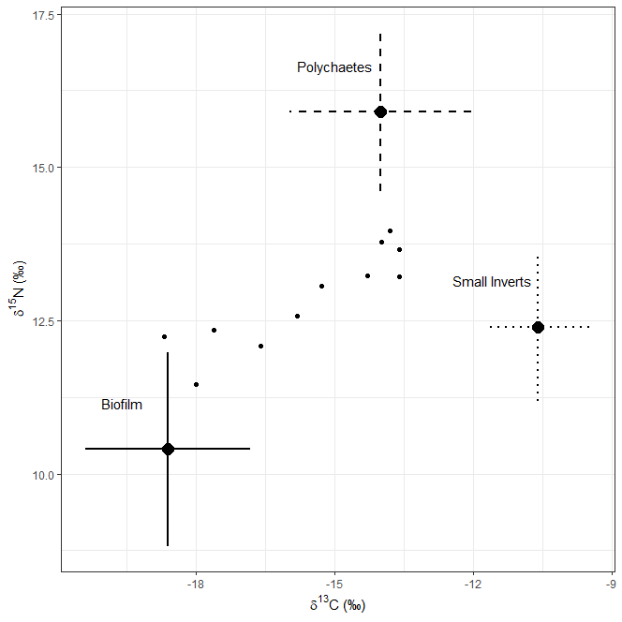

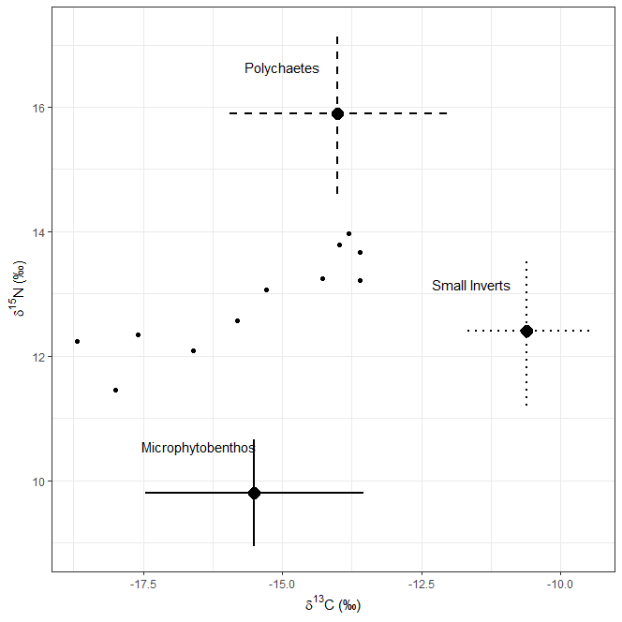

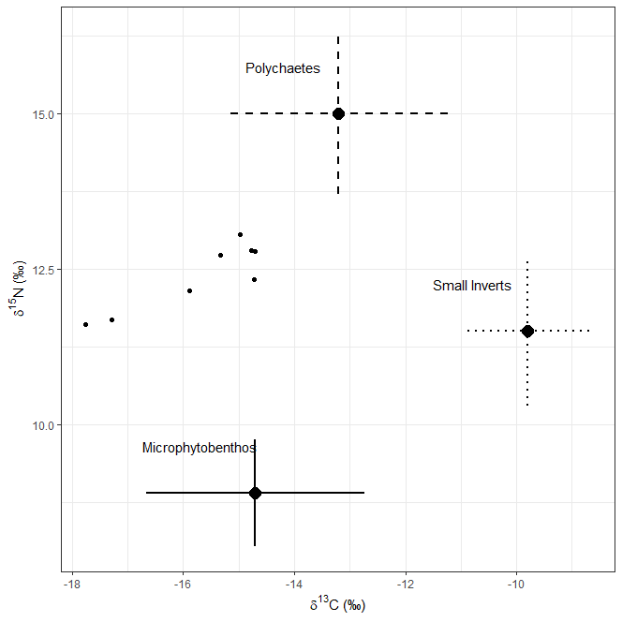


Figure S2. Posterior probability distributions of dietary inputs to A) Western Sandpiper and B) Dunlin based on muscle δ^13^C and δ^15^N measurements and using the MixSiar Bayesian mixing model with either “biofilm” or “microphytobenthos [MPB] as a dietary option. Samples were from staging shorebirds during 20-23 April 2017 and 2019.


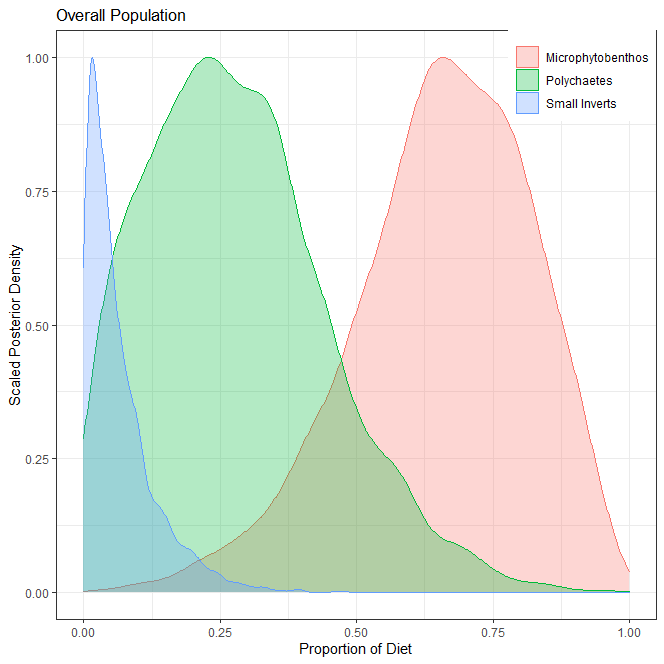

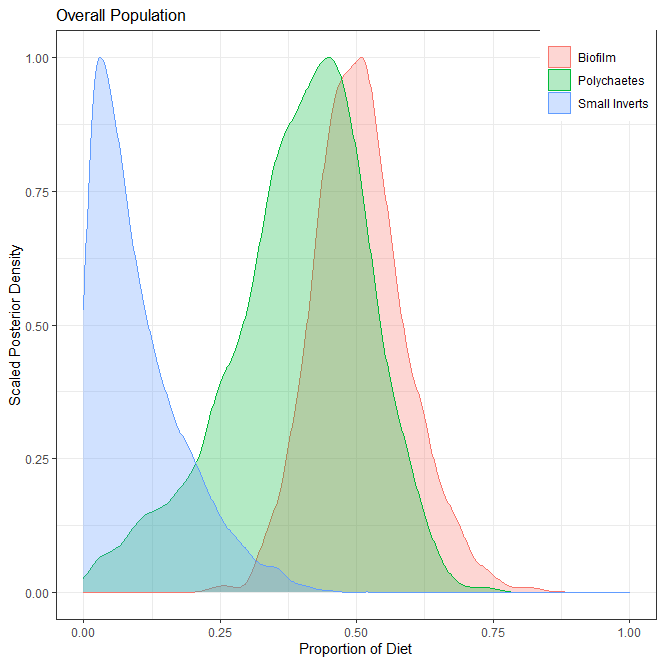

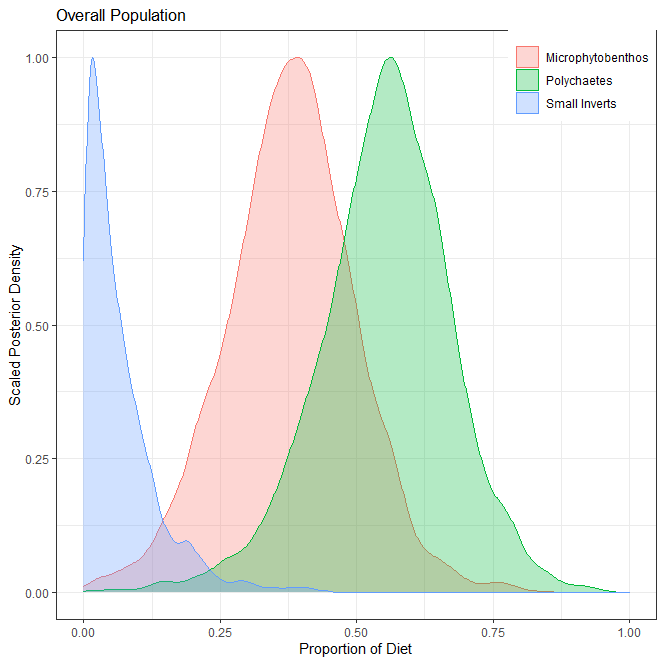

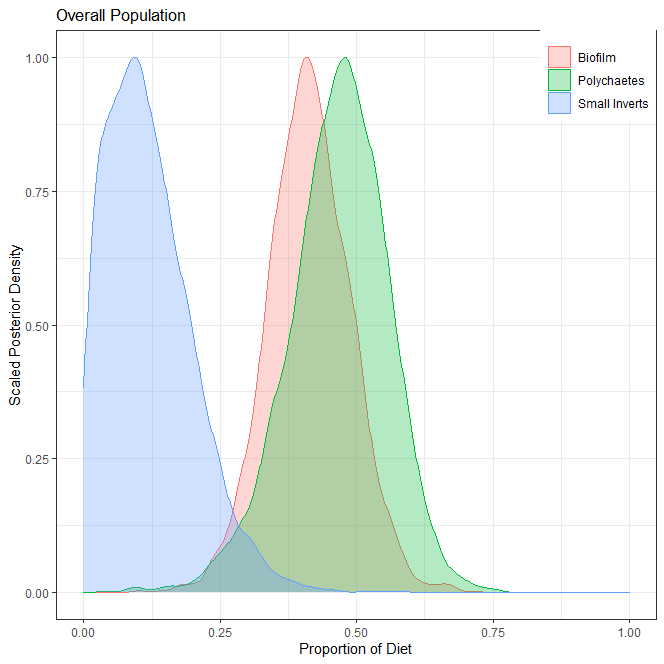


WESA-MPB

WESA-BIOF

DUNL-MPB

DUNL-BIOF
